# Adverse Drug Reactions and Drug Interactions in Multimorbid Patients: A Review of Current Evidence

**DOI:** 10.7759/cureus.97640

**Published:** 2025-11-24

**Authors:** Harsh S, Shailesh Tripathi, Rakesh Venuturumilli, Shanmuga Priya K, G Harsha Vardhan Reddy, Bikramaditya Mukherjee, Hairya Ajaykumar Lakhani

**Affiliations:** 1 Forensic Medicine and Toxicology, Teerthanker Mahaveer Medical College and Research Centre, Moradabad, IND; 2 Hospital Administration, Rajendra Institute of Medical Sciences, Ranchi, IND; 3 Critical Care and Anesthesia, NASA Hospitals, Hyderabad, IND; 4 Respiratory Medicine, Faculty of Medicine, Sri Lalithambigai Medical College and Hospital, Dr. M.G.R. Educational And Research Institute, Chennai, IND; 5 HPB Surgery and Liver Transplantation, Institute of Liver and Biliary Sciences, New Delhi, IND; 6 Biochemistry, KPC Medical College, Kolkata, IND; 7 Internal Medicine, SBKS Medical College and Research Center, Vadodara, IND

**Keywords:** adverse drug reactions, deprescribing, drug-drug interactions, geriatric pharmacotherapy, multimorbidity, polypharmacy

## Abstract

The rising prevalence of multimorbidity, defined as the coexistence of two or more chronic health conditions, is particularly pronounced among older adults and often necessitates polypharmacy. This increases the risk of adverse drug reactions (ADRs) and drug-drug interactions (DDIs), yet multimorbid individuals remain underrepresented in clinical trials. This narrative review synthesizes evidence from 55 recent sources, including randomized controlled trials, observational studies, systematic reviews, and real-world data, to evaluate the burden, risk factors, and management strategies for ADRs and DDIs in this vulnerable population. The findings highlight that ADRs and DDIs contribute substantially to hospitalizations, therapeutic failures, and morbidity. Common ADRs include gastrointestinal bleeding, hypoglycemia, nephrotoxicity, and central nervous system depression, while high-risk interactions frequently involve drugs such as warfarin, non-steroidal anti-inflammatory drugs (NSAIDs), angiotensin-converting enzyme (ACE) inhibitors, and selective serotonin reuptake inhibitors (SSRIs). Key areas of focus include geriatric multimorbidity, organ dysfunction, and the clustering of comorbidities. The review underscores the utility of deprescribing protocols, electronic decision support systems, and interdisciplinary care approaches. Persistent challenges, such as inappropriate prescribing and the complexity of polypharmacy, call for targeted interventions, including personalized dosing, the incorporation of artificial intelligence for risk prediction, and long-term pharmacovigilance frameworks. Advancing safer pharmacotherapy in patients with multiple conditions (multimorbid patients) requires holistic, evidence-informed, and patient-centered strategies.

## Introduction and background

Multimorbidity, defined as the coexistence of two or more chronic diseases in an individual, has become a critical global health concern in the context of an aging population [1]. Unlike comorbidity, which centers around a dominant index disease with accompanying secondary conditions, multimorbidity represents the presence of multiple chronic illnesses without hierarchy, requiring integrated and patient-centered care approaches [2]. It is particularly prevalent among older adults, where up to 65-98% of individuals aged 65 years or above in high-income nations are affected [3]. The growing number of multimorbid patients worldwide has challenged the disease-specific orientation of traditional healthcare systems and demands comprehensive therapeutic strategies that address combined disease effects rather than isolated pathologies [4].

Among the various multimorbid patterns, cardiometabolic multimorbidity comprising hypertension, diabetes mellitus, and dyslipidemia represents one of the most prevalent and clinically challenging constellations globally. This pattern is frequently observed in older adults and is associated with an elevated risk of adverse drug reactions (ADRs) and drug-drug interactions (DDIs) due to overlapping pharmacotherapies [5]. The concurrent use of antihypertensives, oral hypoglycemics, and lipid-lowering agents (such as statins) can lead to pharmacodynamic (PD) potentiation, hepatotoxicity, electrolyte imbalance, and hypoglycemia when not adequately monitored [6]. Clinically, the consequences of ADRs and DDIs are profound, with bleeding episodes linked to anticoagulant combinations, hypoglycemia associated with insulin or sulfonylureas, and nephrotoxicity from certain drug regimens in patients with renal impairment [7]. These examples underscore the urgency of improving medication safety and adopting coordinated, integrated management approaches in multimorbid populations [8].

Physiological aging, polypharmacy, and complex therapeutic regimens further heighten susceptibility to medication-related harm. Age-related changes, including reduced hepatic and renal function, decreased hepatic blood flow, and diminished cytochrome P450 enzyme activity, impair drug metabolism and clearance [9]. Additionally, altered body composition characterized by a higher fat-to-lean mass ratio and reduced total body water affects drug distribution, while receptor-level alterations and frailty increase PD sensitivity [10]. These factors, together with inappropriate prescribing, illness clustering, and drug-nutrition interactions, significantly contribute to the risk of ADRs and DDIs in older adults [11,12]. In cardiometabolic multimorbidity, for instance, co-administration of renin-angiotensin system inhibitors with potassium-sparing diuretics increases hyperkalemia risk, while beta-blockers with insulin or sulfonylureas may mask hypoglycemic symptoms [13]. Similarly, statins used alongside macrolides or antifungals can trigger rhabdomyolysis due to CYP3A4-mediated inhibition [14].

A high-risk environment for medication-related complications is therefore created in older adults by the combination of physiological ageing, multiple chronic conditions, and extensive pharmacotherapy [15]. Recent evidence by Osanlou et al. highlights that polypharmacy in multimorbid adults is significantly associated with ADR-related hospitalizations, reinforcing the need for proactive pharmacovigilance and personalized prescribing [2]. Hence, polypharmacy and the potential hazards associated with it are integral to multimorbidity management [16]. This review adopts cardiometabolic multimorbidity as the central framework for examining ADRs and DDIs because it exemplifies the complex pharmacotherapy challenges seen across other multimorbid conditions.

Objectives of the review

This review aims to provide a comprehensive examination of ADRs and DDIs in multimorbid individuals, with a specific focus on cardiometabolic multimorbidity as a representative model. It seeks to explore the mechanisms underlying ADRs and DDIs in multimorbid populations, identify the clinical and systemic factors contributing to these risks, and present evidence-based strategies for their prevention, detection, and management. The overarching goal is to inform healthcare professionals on optimizing pharmacotherapy, enhancing patient safety, and addressing the challenges posed by polypharmacy in complex, multimorbid patients.

Methodology

This narrative review synthesizes evidence primarily from studies published between 2015 and 2025, identified through electronic databases, including PubMed, Scopus, and ScienceDirect. Although the design of this review is narrative, a structured and transparent search process was employed to enhance rigor and reproducibility. The literature search used combinations of the keywords “multimorbidity”, “polypharmacy”, “adverse drug reactions,” “drug-drug interactions”, “older adults”, and “pharmacovigilance” linked through Boolean operators (AND, OR).

The search strategy was limited to peer-reviewed articles written in English and focused on studies involving adults aged 18 years or older with two or more chronic conditions. Systematic reviews, meta-analyses, randomized controlled trials, and large observational studies relevant to multimorbidity, ADRs, and DDIs were prioritized. Exclusion criteria included conference abstracts, editorials, commentaries, and case reports with insufficient methodological detail. Additional references were identified through manual screening of the bibliographies of key studies to ensure comprehensive coverage of recent findings and relevant clinical perspectives.

The selection process involved independent screening by two reviewers to minimize selection bias, and discrepancies were resolved through discussion and consensus. Risk of bias was indirectly controlled by including studies with clearly defined methodologies and outcomes related to ADRs and DDIs. Only peer-reviewed journal articles that met these criteria were included to ensure the quality and contemporary relevance of evidence. This approach allowed for a comprehensive yet focused synthesis of data to capture evolving trends in ADRs and DDIs among multimorbid populations.

## Review

The burden of ADRs and DDIs in multimorbid patients extends beyond individual clinical consequences, representing a growing global public health concern [17-19]. Systematic reviews estimate that ADR-related hospitalizations occur in approximately 6-12% of older hospitalized adults, with higher incidences observed among those with multiple comorbidities [20,21]. A cross-sectional study from China further reported a self-reported ADR prevalence of 21% in elderly multimorbid patients, significantly associated with polypharmacy and renal dysfunction [22-24]. The most frequently reported ADRs included gastrointestinal disturbances, dizziness, and cutaneous reactions, primarily resulting from antihypertensives, antidiabetics, and antibiotics [25]. These findings highlight that renal impairment and concurrent use of multiple medications markedly increase susceptibility to drug-related toxicity in older multimorbid adults [26]. Similarly, Osanlou et al. demonstrated that polypharmacy was strongly associated with ADR-related hospital admissions in multimorbid adults, underscoring the close link between medication load and hospitalization risk [2].

Across different regions, the magnitude of the problem varies but remains consistently significant. In Europe, the annual costs attributable to ADRs are estimated to reach up to €79 billion, primarily due to preventable hospitalizations of older multimorbid patients [27]. In North America, ADR-related hospitalizations account for approximately 5-10% of all acute admissions among older adults, with an estimated economic burden exceeding USD 30 billion annually [28]. In contrast, Asian studies demonstrate slightly higher clinical incidence: in India, ADR prevalence among hospitalized older adults is reported at 12.8%, while, in China, it reaches 21% [29]. Similarly, in sub-Saharan Africa, the prevalence ranges between 8% and 15%, depending on healthcare infrastructure and pharmacovigilance systems [30]. Evidence from Latin American countries such as Brazil and Chile indicates that 10-14% of emergency hospitalizations in older adults are drug-related, with more than half classified as preventable [31,32]. This global variability highlights that, although ADR and DDI prevalence are universally high, their recognition and prevention are heavily influenced by regional healthcare capacity, surveillance mechanisms, and access to medication review systems [33,34]. Collectively, regional analyses from North America [27], Europe [26], Asia [24], and emerging data from Latin America [33] and Africa [32] confirm that ADRs and DDIs are a universal yet unevenly controlled health burden.

From a statistical standpoint, pooled meta-analyses indicate that approximately one in 10 hospital admissions worldwide involves an ADR, and nearly half of these events occur in patients prescribed five or more medications [34]. Mortality associated with ADRs in hospitalized elderly patients ranges from 0.2% to 3%, reflecting their considerable clinical severity [35]. Moreover, comparative meta-analytic data reveal that ADR-related mortality and hospitalization rates in low- and middle-income countries are nearly double those of high-income settings, largely due to delayed detection, fragmented follow-up, and limited electronic prescribing systems. These findings establish ADRs and DDIs as a quantifiable, measurable, and globally distributed risk, with both clinical and economic consequences transcending income and geography.

Beyond direct healthcare costs, ADRs and DDIs in multimorbid populations contribute to extended recovery periods, loss of independence, and increased caregiver burden [28]. Their indirect societal costs, such as disability, productivity loss, and long-term care dependency substantial yet underreported, particularly in low-resource regions. From a global health perspective, multimorbidity and associated ADRs disproportionately affect frail and elderly populations, particularly in regions with aging demographics, such as Europe, North America, and East Asia [29]. However, recent evidence from Asia Pacific, Latin America, and sub-Saharan Africa confirms that ADRs and DDIs are also rising rapidly in developing nations, driven by increased access to medications, limited clinical monitoring, and the expansion of chronic disease management programs [31-34]. These populations not only utilize greater healthcare resources but also experience worse outcomes, including functional decline, reduced quality of life, and higher mortality [30]. Understanding the global epidemiology and burden of ADRs and DDIs underscores the urgency of adopting universal pharmacovigilance systems, structured deprescribing protocols, and multidisciplinary care frameworks. In particular, integrating geriatric-focused care models and region-specific surveillance mechanisms can ensure that pharmacological decisions are individualized according to comorbidity patterns, functional capacity, and healthcare setting realities.

Pathophysiology and risk factors

Given the substantial global burden and variation in ADR and DDI prevalence, it is essential to understand the underlying biological and clinical mechanisms that predispose multimorbid patients to these complications.

Pharmacokinetic and Pharmacodynamic Alterations in Aging

The PK and PD of drugs are strongly influenced by age-related physiological changes, which increase the susceptibility of older multimorbid adults to ADRs and DDIs [21,22]. From a PK standpoint, aging is characterized by reduced renal and hepatic clearance, where a decline in glomerular filtration rate (GFR) leads to the accumulation of renally excreted drugs, such as aminoglycosides, digoxin, and metformin, resulting in potential toxicity [23]. Decreased hepatic blood flow, along with reduced activity of multiple hepatic enzyme systems, impairs drug metabolism in older adults. Key enzyme families affected include cytochrome P450 (CYP), flavin-containing monooxygenases (FMOs), uridine 5′-diphospho-glucuronosyltransferases (UGTs), and esterases [24]. These age-related alterations slow the metabolism of lipophilic drugs and heighten the risk of systemic drug accumulation. These enzymes play key roles in phase I and phase II biotransformation reactions, and their age-related decline impairs the metabolism of several therapeutic classes, such as benzodiazepines, β-blockers, and antidepressants. When combined with polypharmacy, this reduced enzymatic diversity increases the likelihood of metabolic competition and adverse drug interactions, contributing to a higher risk of cumulative drug toxicity in older multimorbid adults [1].

As hepatic metabolism declines with age, these PK changes are accompanied by important PD alterations that further heighten vulnerability to drug-related harm. From a PD perspective, alterations in receptor sensitivity, neurotransmitter reserve, and homeostatic regulation modify the body’s response to centrally acting drugs [22]. For instance, older adults with neurodegenerative conditions, such as Parkinson’s disease, Alzheimer’s disease, and dementia with Lewy bodies, exhibit impaired neurotransmitter synthesis and release, particularly of dopamine, acetylcholine, and serotonin, which lead to exaggerated or unpredictable responses to opioids, benzodiazepines, and antipsychotics [25]. These abnormalities heighten the risk of central nervous system adverse effects such as sedation, confusion, hallucinations, and respiratory depression in older adults. Collectively, these physiological changes form the biological foundation of heightened ADR and DDI risk in geriatric multimorbid populations.

Determinants and risk factors of ADRs and DDIs

Patient-Related Factors

Age remains the strongest non-modifiable determinant of ADR risk [1,4]. Frailty, cognitive impairment, reduced functional reserve, and malnutrition further compromise drug metabolism and adherence, predisposing older adults to medication-related harm [4,20]. Genetic polymorphisms affecting drug-metabolizing enzymes such as CYP2D6 and CYP3A4 also modify individual susceptibility, particularly for cardiovascular, anticoagulant, and psychotropic drugs [7,18].

Medication-Related Factors

The number of concurrent medications (polypharmacy) is one of the most consistent predictors of ADRs. Carollo et al. reported that patients taking more than eight medications had nearly double the risk of hospitalization due to ADRs compared to those on fewer drugs [3,10]. Inappropriate prescribing, such as ignoring contraindications or duplicating therapy, amplifies these risks. Although clinical tools such as the STOPP/START and Beers Criteria are designed to identify such errors, their implementation remains suboptimal in routine practice [6,11].

Disease-Related Factors

The nature and combination of comorbidities influence ADR and DDI likelihood. Complex interactions between chronic diseases (e.g., cardiovascular with endocrine or psychiatric disorders) often require multidrug regimens that elevate interaction risk [9,12]. Renal and hepatic dysfunction further exacerbates drug accumulation and toxicity.

System-Related Factors

Fragmented care systems, lack of communication among providers, and inadequate medication reconciliation across healthcare settings contribute substantially to ADR occurrence [17]. Limited geriatric pharmacology expertise and underuse of deprescribing protocols also remain persistent systemic barriers.

Impact of disease clustering on medication risk

Patients with multimorbidity experience more complex pharmacological challenges than those with isolated comorbid conditions. While comorbidity typically involves a dominant index disease with secondary complications, multimorbidity entails multiple concurrent chronic illnesses that interact dynamically, amplifying the risk of ADRs and DDIs. Compared with single-disease management, multimorbid patients often require multidrug regimens targeting independent disease processes, creating overlapping PD and PK effects.

Among the various multimorbidity clusters, cardiometabolic patterns including hypertension, diabetes, and dyslipidemia are the most prevalent and pharmacologically demanding. They require concomitant use of antihypertensives, oral hypoglycemics, and lipid-lowering agents, which can produce metabolic disturbances, electrolyte imbalance, and additive hypotensive or hypoglycemic effects [5,6]. Neuropsychiatric multimorbidity, characterized by depression, anxiety, and cognitive impairment, further compounds medication burden through the addition of psychotropics such as antidepressants and antipsychotics. When combined inappropriately, these drugs may lead to QT prolongation, sedation, serotonin syndrome, or cognitive decline [13,14]. Similarly, oncologic multimorbidity involves polypharmacy with chemotherapeutic and supportive agents such as corticosteroids, antiemetics, and analgesics, which can result in synergistic toxicity, altered drug clearance, and heightened susceptibility to ADRs [8].

Collectively, these patterns demonstrate that the interaction of multiple chronic conditions rather than the presence of any single disease is the principal driver of pharmacological complexity and adverse outcomes. Effective risk mitigation, therefore, requires structured medication review, multidisciplinary coordination, and geriatric-centered pharmacotherapy to enhance safety and therapeutic precision [17,25]. Table 1 summarizes the key multimorbidity clusters and their pharmacological risks.

**Table 1:** Impact of Disease Clustering on Medication Risk in Multimorbid Patients

Multimorbidity Cluster	Common Diseases	Typical Drug Classes Used	Potential Pharmacological Risks	Examples of ADRs/DDIs	Clinical Implications / Management Approaches	References
Cardiometabolic	Hypertension, diabetes mellitus, dyslipidemia	Antihypertensives, oral hypoglycemics, statins	Overlapping metabolic pathways and additive pharmacodynamic effects	Hypotension, hypoglycemia, electrolyte imbalance, hepatic enzyme competition	Regular monitoring of blood pressure, glucose, and electrolytes; rational combination therapy; use of interaction-checking software	[2,7,21]
Neuropsychiatric	Depression, anxiety, cognitive impairment, Parkinson’s disease	Antidepressants, antipsychotics, anxiolytics, dopaminergic agents	CNS depression, altered neurotransmitter activity, pharmacokinetic interference	Sedation, QT prolongation, serotonin syndrome, confusion	Periodic mental status evaluation, dose titration, ECG monitoring, avoidance of duplicate CNS depressants	[1,12]
Oncologic	Solid and hematologic malignancies with comorbid chronic illnesses	Chemotherapeutics, corticosteroids, antiemetics, analgesics	Enzyme inhibition and induction affecting drug clearance; additive toxicity	Myelosuppression, hepatotoxicity, nausea, drug accumulation	Oncology-pharmacy collaboration, medication reconciliation, supportive therapy optimization	[3,8,25]
Geriatric / Frailty-Linked	Osteoarthritis, dementia, chronic kidney disease, heart failure	Analgesics, cognitive enhancers, diuretics, ACE inhibitors	Reduced hepatic and renal clearance, altered distribution and receptor sensitivity	Delirium, falls, dehydration, renal toxicity	Comprehensive geriatric assessment, deprescribing, renal dose adjustments, caregiver education	[1,4,13]

Mechanistic insights into ADRs and DDIs

The mechanisms underlying ADRs and DDIs in multimorbid patients are complex, reflecting the interplay between PK, PD, and patient-specific factors. Age-related decline in renal clearance is one of the most important contributors, predisposing patients to drug accumulation and toxicity. Renally excreted agents such as digoxin, aminoglycosides, and metformin may reach toxic levels in older adults even in the absence of overt kidney disease [31]. Similarly, reduced hepatic blood flow and altered cytochrome P450 (CYP450) enzyme activity, particularly involving CYP2D6 and CYP3A4, impair the metabolism of lipophilic drugs, raising the risk of toxicity in psychotropics, anticoagulants, and cardiovascular medications [32]. PD mechanisms are equally critical. Frailty and diminished homeostatic reserve make older multimorbid patients more susceptible to central nervous system depression from benzodiazepines, opioids, and antipsychotics, often resulting in delirium, falls, and impaired mobility [33]. Comorbid conditions such as cardiovascular disease or psychiatric disorders introduce additional prescribing complexity, where additive effects of multiple agents (e.g., QT prolongation with SSRIs and antipsychotics) can have life-threatening consequences [34].

Beyond PK/PD, drug-nutrient and drug-disease interactions further exacerbate medication risk. Malnutrition and altered protein binding in frail elderly individuals can modify drug absorption and distribution, while alcohol use disorder has been associated with amplified central nervous system-related ADRs in multimorbid patients [35]. Genetic polymorphisms in CYP450 isoenzymes and drug transporters also contribute significantly to interindividual variability, with implications for drugs such as warfarin and antidepressants. Pharmacogenomic testing, although still underutilized in routine practice, has demonstrated the potential to individualize therapy and reduce ADRs, particularly in patients with renal or hepatic impairment [36]. Taken together, these mechanistic insights demonstrate that multimorbidity amplifies the inherent risks of pharmacotherapy through cumulative alterations in drug handling, receptor sensitivity, and patient frailty. Recognition of these mechanisms is essential to tailoring safer treatment regimens, guiding monitoring strategies, and informing personalized approaches to prescribing.

ADRs in multimorbid patients

In multimorbid populations, the interaction between disease burden and pharmacotherapy often leads to clinically significant ADRs such as anticoagulant-related bleeding or hypoglycemia in older diabetic patients [24,25].

Prevalence and Burden

ADRs are markedly more prevalent in multimorbid patients than in those with single conditions, reflecting the cumulative effects of polypharmacy and physiological decline. A systematic review reported that 6-12% of hospitalizations in older adults were directly related to ADRs, with a higher incidence observed in those prescribed multiple long-term medications [23]. A multicenter analysis by Fahmi et al. further demonstrated that complex polypharmacy significantly increased emergency readmission risk within 30 days, highlighting the cyclical impact of ADRs on healthcare utilization [28].

Globally, ADR-related hospitalizations impose a substantial economic burden, with annual costs estimated at €79 billion in the European Union and over USD 30 billion in North America, most attributed to preventable medication-related admissions among elderly multimorbid patients [29]. These figures emphasize that ADRs are not only a clinical issue but also a critical determinant of healthcare resource strain, especially in aging populations with multiple chronic conditions [30].

Most Common ADRs and Implicated Drugs

The range of ADRs experienced by multimorbid patients is wide, although a few patterns can be identified based on population-wide research. GI bleeding is one of the most common ADRs that are usually reported, and this side effect is often related to the intake of non-steroidal anti-inflammatory drugs (NSAIDs), antiplatelet, and oral anticoagulants. In the case of co-prescription of such agents as aspirin with clopidogrel or warfarin, the risk of bleeding becomes high, especially among patients with peptic ulcer disease or chronic kidney disease (CKD) [31,32]. Another serious issue is hypoglycemia, particularly in older patients who use insulin or sulfonylureas [33]. Multimorbidity, including cognitive impairment, malnutrition, and renal dysfunction, increases the risk of severe hypoglycemic events with occasional hospitalization or death [26,34]. Drug-induced nephrotoxicity, including that caused by aminoglycosides, radiocontrast agents, and some chemotherapeutics, is especially hazardous in patients with underlying renal impairment, a frequent comorbidity in the elderly [27]. Combined CNS depressants may cause severe functional deficits, the risk of falls, and institutionalization [30,33].

Psychotropic agents also contribute significantly to adverse outcomes. According to Carollo et al., antipsychotics and antidepressants were among the medications that caused a high percentage of preventable ADRs in older inpatients, especially when used without monitoring or in combination with other drugs [3]. These high-risk medications are common among multimorbid patients, which is why systematic medication reviews and active deprescribing policies are needed, particularly in such a polypharmacy-prone field as geriatrics or oncology [33,35]. These ADRs primarily affect the gastrointestinal, renal, central nervous, and cardiovascular systems and are usually related to high-risk classes of drugs. Table 2 shows the most common ADRs as well as their clinical characteristics, drug classes involved, and patient subgroups most susceptible. The table also includes important literature pointing out the importance of every ADR in clinical practice.

**Table 2:** Common ADRs in Multimorbid Patients: Mechanisms, Clinical Manifestations, and Implicated Drug Classes ADRs: Adverse drug reactions; CKD: Chronic kidney disease; NSAIDs: Nonsteroidal anti-inflammatory drugs; SSRIs: Selective serotonin reuptake inhibitors

ADR Type	Clinical Manifestation	Drug Classes Involved	Risk Factors	Patient Population at Risk	Ref.
GI bleeding	Melena, hematemesis	NSAIDs, anticoagulants	Age, CKD	Elderly, CV comorbidities	[31,32]
Hypoglycemia	Confusion, dizziness	Insulin, sulfonylureas	Frailty	Elderly diabetics	[25,26,34]
Nephrotoxicity	Increased serum creatinine	Aminoglycosides, contrast agents	CKD	Oncologic/elderly	[27]
CNS depression	Sedation, delirium	Benzodiazepines, opioids	Polypharmacy	Frail geriatric	[30,33]
QT prolongation	Palpitations, syncope	SSRIs, antipsychotics	Electrolyte imbalance	Psychiatric multimorbidity	[36]
Hyperkalemia	Arrhythmias	ACEi + K-sparing diuretics	CKD	HF patients	[35]
Falls	Fractures, bruising	Sedatives, antihypertensives	Orthostatic hypotension	Geriatric/frail patients	[31]
Hepatotoxicity	Elevated LFTs	Statins, antiepileptics	Hepatic impairment	Oncology, epilepsy patients	[30]
Serotonin syndrome	Fever, tremors, agitation	SSRI + MAOI/Triptans	Polypharmacy	Psychiatric polytherapy	[34]
Drug-induced delirium	Acute confusion	Anticholinergics, opioids	Cognitive impairment	Elderly	[37]

Drug-Drug Interactions (DDIs)

DDIs occur when the pharmacological effect or concentration of a drug is altered by the presence of another. These interactions are broadly classified as PK or PD. PK interactions involve changes in drug absorption, distribution, metabolism, or excretion. The cytochrome P450 (CYP) enzyme family-particularly CYP3A4 and CYP2D6-plays a key role in hepatic drug metabolism. These enzymes may be inhibited or induced by other agents, leading to clinically significant variations in plasma drug concentrations. For instance, CYP3A4 inhibitors such as certain macrolide antibiotics and antifungals can elevate plasma levels of co-administered drugs, causing toxicity [31,32]. Conversely, enzyme inducers such as carbamazepine or rifampicin accelerate the metabolism of agents such as oral contraceptives or antiretrovirals, reducing their therapeutic effect.

Such metabolic interactions are further influenced by genetic polymorphisms that alter enzyme expression, particularly in older adults with polypharmacy [33]. PD interactions, on the other hand, occur when drugs exert additive, synergistic, or antagonistic effects on shared targets at the receptor, organ, or systemic level. These can arise even within therapeutic concentrations. For example, co-administration of selective serotonin reuptake inhibitors (SSRIs) and nonsteroidal anti-inflammatory drugs (NSAIDs) increases gastrointestinal bleeding risk by impairing platelet aggregation and mucosal protection [34]. Similarly, additive effects between antihypertensives and diuretics can predispose to hypotension or electrolyte imbalance, while concurrent anticoagulant and antibiotic use may elevate bleeding risk by altering vitamin K synthesis [35]. Understanding these mechanisms provides the foundation for recognizing clinically important DDIs that frequently occur in multimorbid patients.

Clinical Examples of High-Risk DDIs

Mechanistic insights translate directly into several clinically significant interactions seen in multimorbid populations. One of the most critical is the combination of warfarin with antibiotics such as metronidazole, macrolides, or fluoroquinolones, which interferes with warfarin metabolism and gut flora, raising the international normalized ratio (INR) and bleeding risk [34]. Another frequent concern is hyperkalemia resulting from the concomitant use of ACE inhibitors and potassium-sparing diuretics, especially in patients with renal impairment [35]. Similarly, SSRIs combined with NSAIDs markedly increase the risk of upper gastrointestinal bleeding when gastroprotective measures are absent [34].

These high-risk DDIs emphasize the necessity of individualized, patient-centered management. Regular monitoring of renal function, electrolytes, and coagulation parameters, coupled with periodic medication reviews, can prevent serious complications in older or frail patients with complex drug regimens.

Polypharmacy and the Risk of DDIs

As the number of prescribed drugs increases, the likelihood of DDIs escalates exponentially. The probability of an interaction is estimated at 13% with two drugs, rising to 58% with five drugs and over 80% with seven or more [36]. This risk is amplified in multimorbid patients managed by multiple healthcare providers, where fragmented prescribing often produces uncoordinated treatment plans. Network analyses of cardiovascular, metabolic, and psychiatric medication regimens reveal intricate interaction networks involving commonly used agents such as anticoagulants, antihypertensives, antipsychotics, and antidepressants [37].

In the absence of pharmacist oversight or electronic support systems, these interactions frequently go undetected in clinical settings. Robinson et al. highlighted that polypharmacy among older adults should not be judged merely by the number of drugs prescribed, but by their appropriateness, potential interactions, and cumulative functional effects [30]. Complex polypharmacy involving CNS-active and cardiovascular agents, therefore, warrants systematic review and ongoing monitoring to minimize preventable harm.

Clinical and system-level implications

Diagnostic Challenges

ADRs and DDIs in multimorbid patients are challenging to diagnose, as their symptoms often overlap with those of chronic illnesses, leading to diagnostic overshadowing. For example, dizziness caused by antihypertensives may be misattributed to anemia or Parkinson’s disease, while cognitive changes due to anticholinergics may mimic dementia progression [38]. These challenges are further intensified by communication barriers, poly-symptomatology, and cognitive decline, which delay recognition and prolong exposure to harmful medications [39]. Additionally, excessive electronic alerts can induce clinician “alert fatigue,” causing critical warnings to be overlooked. This highlights the need for intelligent, context-sensitive diagnostic support systems that prioritize clinically meaningful interactions.

Therapeutic and Management Implications

Unrecognized ADRs and DDIs often trigger prescribing cascades, where additional drugs are introduced to manage side effects rather than addressing their cause. This reinforces polypharmacy, complicates titration, and heightens vulnerability in older adults with reduced physiological reserve. Interruptions in therapy due to unaddressed ADRs may also lead to disease destabilization, treatment non-adherence, and functional decline. A structured, proactive approach emphasizing medication reconciliation, risk stratification, and deprescribing can help mitigate these outcomes.

System-Level and Economic Implications

At the healthcare-system level, ADRs and DDIs are major drivers of preventable hospitalizations, prolonged hospital stays, and readmissions, placing financial pressure on already strained health budgets. These events divert critical resources from preventive and rehabilitative care. Implementing integrated medication review protocols, interprofessional communication frameworks, and real-time pharmacovigilance systems can substantially reduce this burden. Broader investments in clinical decision-support technologies and training in geriatric pharmacotherapy remain essential for sustainable improvement in medication safety.

Clinical management framework for ADRs and DDIs

The management of ADRs and DDIs in multimorbid patients demands a structured, anticipatory framework that prioritizes early recognition and coordinated intervention. Addressing diagnostic overshadowing requires comprehensive medication histories, assessment of temporal relationships, and active evaluation for drug-disease and DDIs.

A stepwise management strategy is most effective. First, conduct comprehensive medication reconciliation, especially for patients seeing multiple providers, to identify duplicate or high-risk prescriptions [38]. Second, differentiate ADRs from disease progression using validated tools such as STOPP/START (Screening Tool of Older Person's Prescriptions/Screening Tool to Alert to Right Treatment) or Beers Criteria, supported by clinical and PK monitoring. Third, employ a hierarchical intervention approach: (i) deprescribe non-essential or high-risk drugs, (ii) substitute safer alternatives when appropriate, and (iii) tailor doses according to renal, hepatic, and frailty status [39].

Integrated multidisciplinary collaboration among physicians, pharmacists, and nurses ensures individualized therapy and continuous reassessment as patient conditions evolve. For instance, warfarin co-prescribed with fluoroquinolones substantially increases bleeding risk due to altered metabolism and gut flora, requiring close INR monitoring or substitution with safer antibiotics [40]. Similarly, combined ACE inhibitor and potassium-sparing diuretic therapy necessitates proactive electrolyte monitoring to prevent hyperkalemia [41].

Emerging technologies, including EHR-integrated Clinical Decision Support Systems (CDSS) and AI-driven predictive models, now enable real-time DDI detection and ADR risk assessment [42]. When combined with pharmacist-led interventions and geriatric expertise, these tools form the foundation of an intelligent, data-informed pharmacotherapy ecosystem that enhances medication safety, continuity of care, and patient outcomes [43].

Medication review and deprescribing

One of the mainstays of DDI and ADR prevention is medication review. Structured techniques such as the Beers Criteria and the STOPP/START criteria are used to identify potentially inappropriate medications (PIMs) in older individuals [40]. Particularly helpful are the techniques for spotting high-risk medicines, such as anticholinergics, long-acting benzodiazepines, and duplication treatments. Recently, a systematic literature review conducted in Italy highlighted the success of medication reviews conducted by pharmacists in lowering the incidence of PIMs and avoiding ADR-related hospitalization [35]. Furthermore, specific interventions in such facilities as long-term care and oncology have proved that deprescribing not only decreases polypharmacy but also leads to improved clinical outcomes, including cognitive clarity, mobility, and the quality of life [33,41]. Deprescribing is an organized procedure that seeks to reduce or withdraw medications when the risks are greater than the benefits. It involves a close assessment of patient objectives, life span, and the likelihood of withdrawal. Examples include the reduction of adverse outcomes in patients with orthostatic hypotension and falls, and falls with deprescribing antihypertensives in cardiology, which does not affect cardiovascular health [41].

Deprescribing protocols led by pharmacists, such as the one described by Raju et al. in oncology, were shown to be able to identify in more than 60% of patients unnecessary medications and decrease the burden of the regimen without having an adverse impact on disease control [25,42]. The primary tool for lowering the probability of ADRs as well as DDIs in the setting of polypharmacy is structured medication review techniques. Numerous models and tools have been evaluated in various care settings, ranging from computerized decision support systems to clinical criteria. Table 3 gives a summary of major interventions, populations they are aimed at, and the evidence of their effectiveness, as well as the literature in which these strategies were tested.

**Table 3:** Clinical Decision Tools and Risk Mitigation Strategies for ADRs and DDIs in Multimorbid Patients ADRs: Adverse drug reactions; CDSS: Clinical Decision Support Systems; DDIs: Drug-drug interactions; STOPP/START: Screening Tool of Older Person's Prescriptions/Screening Tool to Alert to Right Treatment

Tool/Strategy	Description	Target Group	Setting	Evidence of Effectiveness	Ref.
STOPP/START Criteria	Identify inappropriate prescriptions	Older adults	Geriatrics, primary care	Reduces ADRs, PIMs	[40]
Beers Criteria	Guide for potentially inappropriate meds	65+ years	All care levels	Validated for geriatrics	[40]
CDSS	Real-time alert for DDIs, renal dosing	All multimorbid patients	EHR-integrated	Preventable ADR detection	[42,43,44]
Pharmacist-led Review	Medication audit by pharmacists	Polypharmacy patients	Hospital, LTCF	Improves adherence, reduces PIMs	[35,41]
Deprescribing Protocols	Taper/discontinue non-beneficial drugs	Frail elderly	Oncology, geriatrics	Better cognition/mobility	[33]
Trigger Tools (e.g., TRIGGER-CHRON)	Surveillance for high-alert drugs	The elderly with multimorbidity	Hospitals	Increases ADR reporting	[15]
Polypharmacy Guidelines (ESC)	Cardio-pharma risk reduction	CV multimorbidity	Cardiology clinics	Decrease DDI frequency	[45]
AI/ML Risk Prediction	Predictive ADR detection models	High-risk polypharmacy	Research/EHR	Prototypes in development	[46]
Pharmacogenomic Testing	Dose adjustment via CYP profile	Renal/hepatic impairment	Personalized care	Implementation studies ongoing	[40]
Interdisciplinary Rounds	Combined physician–pharmacist review	Long-term patients	Geriatrics	Reduces medication errors	[33]

Clinical Decision Support Systems (CDSS)

CDSS have become common in electronic health records (EHRs) to help clinicians deal with complicated medication regimens. These systems provide real-time alerts on DDIs, renal dose adjustments, and duplicate therapy risks [42]. More sophisticated CDSSs use patient-specific values, including age, comorbidities, and laboratory values, to decrease the relevance of alerts. To illustrate, incorporation of kidney function data can help avoid nephrotoxic dosing of patients using aminoglycosides or NSAIDs [43]. The quality of these systems is, however, dependent on the quality of the data, the logic of alert algorithms, and user compliance. Recently, Tamargo et al. emphasized that, when CDSS systems are created with specialty-specific modules, they can be invaluable in the management of cardiovascular polypharmacy [45]. On the same note, the study by Gaspar et al. indicated that automated detection tools incorporated into EHRs would considerably decrease avoidable ADRs in geriatric care [46]. Collectively, such systems have demonstrated measurable benefits, including reductions of up to 20-30% in preventable ADR-related hospitalizations and improved adherence to safe prescribing practices, validating their real-world effectiveness in minimizing medication harm. Nevertheless, there are limitations. Excessive alerting may lead to alert fatigue, which makes clinicians numb to critical warnings and disregard or silence them. In such a way, optimization of alert thresholds and incorporation of clinical context are important. In order to achieve maximum utility, CDSS should be combined with training, user-friendly design, and collaboration across the disciplines of pharmacists, physicians, and IT specialists. When properly implemented, the tools enhance medication safety, minimize unnecessary harm, and foster a culture of safety in the prescription process [44,45].

Special populations

Geriatric Multimorbidity

Older adults constitute the largest multimorbid subgroup and face the highest ADR and DDI risk due to frailty, cognitive decline, and PK changes. Even mild ADRs can cause functional decline, while atypical presentations delay diagnosis [46,47]. Commonly implicated agents include benzodiazepines, antihypertensives, and anticholinergics, with CNS effects frequently reported in those with alcohol use disorder [39]. Moreover, atypical or masked clinical presentations in older adults often delay ADR diagnosis [47].

Mental Health Comorbidity

Coexisting psychiatric and physical illnesses add substantial prescribing complexity. Psychotropic agents, such as antipsychotics, antidepressants, and lithium, possess narrow therapeutic ranges and high interaction potential [36,48]. For example, clozapine, used for agitation in dementia, requires close monitoring for agranulocytosis, hypotension, and sedation [49]. Heck et al. found psychiatric polypharmacy to be strongly associated with both type A and type B ADRs [50]. These agents should therefore be used cautiously, with individualized titration and frequent review in patients with organ impairment.

Renal and Hepatic Dysfunction

Renal and hepatic impairment significantly alter drug metabolism and clearance. Aminoglycosides, NSAIDs, and digoxin require careful dose adjustments in renal dysfunction, as confirmed by Wang et al., who found high ADR prevalence among elderly patients with CKD and polypharmacy [26]. Similarly, hepatic impairment affects the metabolism of psychotropic and cardiovascular drugs, necessitating the use of the Child-Pugh or eGFR-based dosing systems [51]. Incorporating pharmacogenomic testing can further optimize dosing and reduce ADRs in individuals with impaired metabolic capacity [40].

Evidence from recent literature

Systematic Reviews and Meta-Analyses

An increasing literature base that includes meta-analyses and systematic reviews has tried to quantify the risks of ADRs and evidence gaps. Systematic reviews consistently link polypharmacy and inappropriate prescribing with increased hospitalization and mortality among older multimorbid adults [35,52]. Lleal et al. found that complex multimorbidity clusters were strongly associated with the use of PIMs [53]. The authors discovered that patients whose chronic disease clusters were more complex were more prone to be prescribed PIMs [53].

Real-World Evidence (RWE)

EHRs, registries, and administrative databases are real-world data that offer essential information about the prevalence and preventability of ADRs. As an example, a study conducted by Zerah et al. based on the OPERAM trial data proved the efficacy of trigger tools to identify drug-related hospitalization, which is not commonly recognized in clinical practice [54]. One more instance is the usefulness of the TRIGGER-CHRON tool in detecting ADRs associated with high-alert drugs in elderly patients with multimorbidity, which highlights the importance of data-driven surveillance approaches [55].

Key Trials and Observational Studies

New randomized controlled trials and cohort studies have been able to add value to our knowledge on medication-related harm. Kempen et al. conducted a post-hoc analysis to show that more than 50% of drug-related hospital revisits in older adults are preventable and that most of the risk is due to medication errors and DDIs [47]. Gaspar et al. proposed and validated CDSS enhanced with machine learning algorithms and integrated within EHR platforms to automatically detect potential ADRs in geriatric patients [41]. This development marks a pivotal step toward intelligent pharmacovigilance, allowing real-time identification of high-risk drug combinations, early prediction of adverse events, and targeted clinical interventions. Such AI-driven systems can substantially reduce diagnostic delays, improve prescribing safety, and support personalized medication management, transforming ADR monitoring from a reactive to a proactive process in multimorbid populations [46]. These revelations support the transition between passive and proactive medication safety.

Strategies for risk mitigation

Addressing medication-related harm in multimorbid patients requires a multifaceted approach that integrates clinical collaboration, patient engagement, and policy-level interventions.

Interdisciplinary Care Models

Due to the complexity of handling the multimorbid patients, an interdisciplinary approach is required, which includes geriatricians, primary care physicians, clinical pharmacists, and mental health professionals. Pharmacist-led structured medication review can help to decrease PIMs, improve adherence, and reduce ADRs [41]. The inclusion of pharmacists in the inpatient and outpatient care teams enables the early detection of high-risk medications, optimization of doses, and organization of deprescribing programs. The collaborative care models are particularly helpful in geriatrics and oncology, where treatment is highly dense and overlapping [33,35].

Patient Education and Engagement

The issue of patient empowerment and shared decision-making plays an important role in the prevention of medication-related harm. The patients should be informed about the indications, possible side effects, and symptoms of ADRs related to their medicines. This is especially significant to the older adults and caregivers who might not naturally understand the distinction between drug effects and disease progression [38]. Research indicates that an active involvement of the patients in the care planning process leads to increased adherence, minimized medication burden, and enhanced health outcomes [49]. The knowledge can be reinforced and safer practices promoted with visual tools, medication calendars, and telemedicine follow-ups.

Policy and Prescribing Guidelines

Guidelines on safe prescribing practices have been published by the international health authorities. The World Health Organization (WHO), the National Institute for Health and Care Excellence (NICE), and the US Food and Drug Administration (FDA) organizations promote the role of evidence-based prescribing, regular medication review, and the use of pharmacogenomics in everyday practice [40,45]. Tamargo et al. also emphasized the role of harmonized polypharmacy guidelines in older age in the context of cardiovascular disease, suggesting the use of European Society of Cardiology recommendations that focus on the concept of individualized care and the prevention of duplication of therapy [45]. Moreover, introducing trigger tools, including those that were validated by Toscano Guzmán et al. [15] and Sandoval et al. [55], may serve as a standardized surveillance measure to detect high-risk drugs and enhance audit activities in hospitals and long-term care facilities. Figure 2 illustrates an integrated model in which patient-focused interdisciplinary medication review is at the center of mitigating ADRs and DDIs.

**Figure 1:**
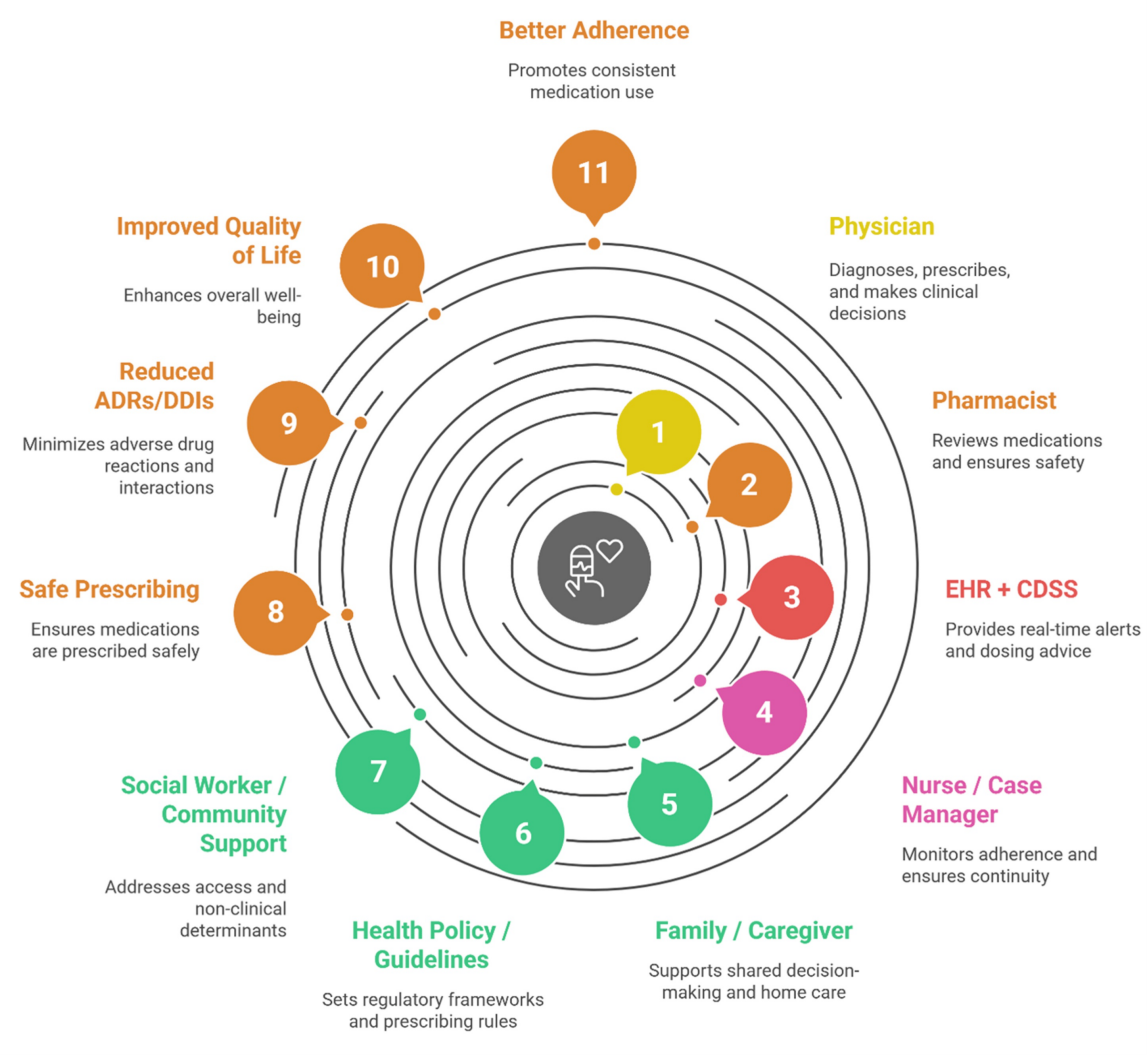
Interdisciplinary Medication Review Model Designed by the author (Harsh S.) using Napkin AI. ADRs: Adverse drug reactions; CDSS: Clinical Decision Support Systems; DDIs: Drug-drug interactions

Policy, guidelines, and future directions

The management of ADRs and DDIs in multimorbid patients is increasingly guided by international policy frameworks and evidence-based clinical guidelines. The WHO, the NICE, and FDA emphasize evidence-based prescribing, structured medication reviews, and the integration of pharmacogenomics into clinical workflows [44]. In cardiovascular medicine, the European Society of Cardiology (ESC) promotes polypharmacy-specific guidance that encourages individualized prescribing, dose optimization, and avoidance of therapeutic duplication - measures shown to reduce clinically significant DDIs [45]. However, translating these global recommendations into everyday practice remains inconsistent due to variations in infrastructure, clinician training, and digital integration capacity. Pharmacogenomic testing is still largely confined to specialized centers, while disparities in healthcare resources hinder equitable access to precision prescribing [46]. The effectiveness of CDSSs is further limited by clinician “alert fatigue,” reinforcing the need for context-sensitive, clinically relevant alerts [47]. Fragmented care delivery, shortages of trained professionals, and limited deprescribing education continue to constrain guideline implementation [48].

Patient and caregiver engagement remains a cornerstone of safe pharmacotherapy, moving beyond passive consent to active participation through shared decision-making, digital adherence tools, and telemonitoring [49]. In populations where multimorbidity overlaps with frailty and cognitive decline, structured caregiver involvement is essential for ensuring safety and continuity of care [50]. Despite progress, important knowledge gaps persist. Most pharmacological trials still exclude multimorbid older adults due to frailty and heterogeneity, limiting the external validity of findings and the creation of representative prescribing guidelines [51]. Current evidence also relies heavily on cross-sectional data; longitudinal studies are required to capture cumulative risks of polypharmacy, the long-term outcomes of deprescribing, and links between ADRs and functional decline [52]. Observational cohorts such as MoPIM have already demonstrated how multimorbidity patterns affect prescribing quality, but sustained data collection is needed to refine dynamic medication management [53].

Looking forward, technological innovation offers the most transformative potential for pharmacovigilance. AI and machine learning models integrated with real-world data from EHRs can predict ADRs and DDIs before clinical manifestation [54]. When combined with pharmacogenomic testing, these systems may usher in a new era of personalized, predictive, and preventive pharmacotherapy [55]. Realizing this vision will require collaboration among researchers, clinicians, policymakers, and digital health innovators to build systems that are both technologically robust and globally accessible. Future strategies must integrate guideline-driven practice, patient-centered engagement, and data-driven technologies to enhance medication safety and effectiveness in multimorbid populations. Bridging existing evidence and implementation gaps demands coordinated international efforts to re-engineer pharmacotherapy for an aging global society [56].

## Conclusions

This discussion shows that the problem of ADRs and DDIs in patients with multiple comorbidities is very complex and multidimensional, especially among older individuals. Polypharmacy, though a longstanding clinical concern, has gained renewed significance with increasing life expectancy and the growing burden of multimorbidity worldwide. Current efforts are therefore directed toward improving medication safety, minimizing ADRs and DDIs, and optimizing individualized pharmacotherapy. However, it greatly augments the risk of preventable drug-related harm, including gastrointestinal hemorrhage, hypoglycemia, nephrotoxicity, and central nervous system depression. The implications of aging on PK and PD, the information about which is in the understanding of the mechanism of change, the existence of complications due to the presence of other diseases of the mental sphere, and organ dysfunction make it clear that one should pay attention to the treatment with medication. Furthermore, the facts presented in systematic research, practical data, and clinical trials prove that the existing measures are not enough to understand the ever-changing character of the dangers polypharmacy presents to these groups. The main lesson in this review is that a whole-pathway patient-centered approach is an urgent requirement that will integrate interdisciplinary teamwork, personalized prescribing, deprescribing, and digital supporting systems. The STOPP/START criteria, clinical decision support systems, and pharmacogenomic profiling tools have high potential but require broader clinical implementation and support. Finally, the fact that multimorbid patients are not well represented in clinical trials and that predictive analytics are absent also means that there is a research gap. There should be a call to action to reengineer clinical practice and generate robust, diverse evidence that safeguards the safety of medications and improves long-term health outcomes of this at-risk group.
